# RNA-mediated gene silencing can reduce azole resistance, growth and pathogenicity in *Pseudocercospora fijiensis*

**DOI:** 10.1371/journal.pone.0325057

**Published:** 2025-06-05

**Authors:** Flor Yuranny Canacuán Melo, Yohana Katerine Suárez Anaya, Vicente Emilio Rey Valenzuela, Juan Gonzalo Morales Osorio, Rafael Eduardo Arango Isaza

**Affiliations:** 1 Escuela de Biociencias, Facultad de Ciencias, Universidad Nacional de Colombia Sede Medellín, Medellín, Antioquia, Colombia; 2 Unidad de Biotecnología Vegetal UNALMED – CIB, Corporación para Investigaciones Biológicas (CIB), Medellín, Antioquia, Colombia; 3 Fitopatología, Cenibanano – Augura, Apartadó, Antioquia, Colombia; 4 Departamento de Ciencias Agronómicas, Facultad de Ciencias Agrarias, Universidad Nacional de Colombia Sede Medellín, Medellín, Antioquia, Colombia; Universitat Jaume 1, SPAIN

## Abstract

The use of double-stranded RNA (dsRNA) gene silencing has allowed significant advances in the understanding of gene function in many organisms and has the potential for use in disease control. At present, there are no adequate methods to induce gene silencing in *Pseudocercospora fijiensis* (synonym *Mycosphaerella fijiensis*), the causal agent of black sigatoka, the most economically important disease affecting banana and plantain production. In this work, we developed methods for effective transient gene silencing in both conidial and mycelial cells of *P. fijiensis.* The results revealed that treatment with small interfering RNA (siRNA) targeting the *PfCyp51* gene was able to reduce the tolerance of a *P. fijiensis* azole-resistant strain to propiconazole. Furthermore, the selective repression of the *Cyp51*, *adenylate cyclase*, and *Fus3* genes resulted in inhibited germ tube and mycelial growth, as well as a decrease in the severity of infection in banana leaves. This study suggests that the analyzed genes may be targets for the control of fungicide resistance in *P. fijiensis* and black Sigatoka disease. RNA interference (RNAi) might be used in the future as a control mechanism in *P. fijiensis* if appropriate delivery methods are found.

## Introduction

Black Sigatoka or Black Leaf Streak, caused by the fungus *Pseudocercospora ﬁjiensis,* is the most economically important disease in bananas and plantains [1,2]. Banana is the second most important fruit worldwide in terms of production and consumption [3]. This disease reduces the photosynthetic leaf area, diminishes a plant’s photosynthetic capacity and adversely affects its growth and productivity. In addition, Black Sigatoka induces premature ripening of fruit bunches, making it unsuitable for commercial use. Without precise and timely management, the disease can completely destroy a plantation [4–6].

Black Sigatoka is controlled mainly through the extensive application of chemical fungicides [7], usually through aerial spraying, which occurs more than 70 times per year in many banana-growing regions [8]. Chemical control has several negative impacts, including environmental effects, human health problems, high control costs, especially for small banana producers, and the emergence of fungicide-resistant *P. fijiensis* populations [4,9,10]. For this reason, black sigatoka management requires new, effective, efficient, and sustainable control methods.

RNA-mediated gene silencing has emerged as a promising technology for crop protection, offering the potential to suppress the expression of key genes in fungal pathogens [11–13]. Approaches such as spray-induced gene silencing (SIGS) and host-induced gene silencing (HIGS) have demonstrated effective disease control in different pathosystems and present promising avenues for further exploration [14,15]. This technology has been employed in several pathogens to target genes associated with growth and pathogenicity [16–20]. In *P. fijiensis*, RNAi has proven to be effective in suppressing genes crucial for its virulence and growth. For example, silencing of the *PfSlt2*, *PfFus3*, and *PfHog1* genes through *Agrobacterium tumefaciens*-mediated transformation significantly reduced their expression levels, resulting in decreased virulence and growth of infected plant tissues with the transformants [21,22]. Similarly, transformants in which *PKS8−2* or *PKS10−2* was silenced were developed and evaluated for pathogenicity. Notably, inoculating banana plants with *PKS10–2*-silenced transformants resulted in a significant reduction in disease symptoms, which was correlated with the level of gene silencing observed in the conidia used for inoculation [23].

Despite its potential, *Agrobacterium*-mediated transformation remains labor intensive, with low success rates, limiting its applicability. While it remains a valuable tool for research, it is not feasible for large-scale disease control in the field. An alternative approach, as reported by Mumbanza, Kiggundu [24], involves the use of synthetic dsRNA molecules targeting adenylate cyclase and DNA polymerase genes, which reduce the in vitro germination of conidia from both *Fusarium oxysporum* and *P. fijiensis*. However, the authors did not quantify gene silencing in their study to confirm the efficacy of this method.

Selecting appropriate target genes within a pathogen and establishing effective, species-specific silencing methods are essential requirements [25]. The polyethylene glycol (PEG)-based transfection method, supplemented with lithium chloride (LiCl), has been shown to offer a simpler and less resource-intensive approach to delivering small interfering RNAs (siRNAs) directly into fungal cells [17]. PEG facilitates the uptake of nucleic acids by fungal cells, whereas LiCl enhances the efficiency of transfection [26]. Although this method has been successfully applied to other fungal species, it holds significant promise as an alternative to fungal transformation for *P. fijiensis*.

Potential target genes for RNA silencing-based management of Black Sigatoka include adenylate cyclase (AC), Fus3, and CYP51. CYP51 encodes an enzyme targeted by sterol demethylation inhibitor (DMI) fungicides, which are widely used for controlling *P. fijiensis* [27,28]. However, the intensive use of DMIs has exerted selective pressure, fostering the emergence of resistant strains [28]. The overexpression of *CYP51* has been identified as a contributing mechanism for this resistance [29], suggesting that reducing its expression could be an effective strategy for resistance management. *Fus3,* which encodes the MAPK (Mitogen-activated protein kinase) protein FUS3, has been studied in several organisms and is necessary for growth and virulence in various phytopathogenic fungi [30,31]. *AC* catalyzes the formation of cyclic adenosine monophosphate (cAMP), a signaling molecule involved in nutrient sensing, stress responses, metabolism regulation, development, differentiation, and sexual reproduction [32,33].

In this study, we adapted and evaluated an in vitro gene silencing method for *P. fijiensis* that does not require fungal transformation. This approach aimed to demonstrate the applicability of gene silencing for studying gene function and identifying potential targets for controlling Black Sigatoka disease. We utilized siRNA sequences targeting *PfAC*, *PfCYP51*, and *PfFus3*, which significantly reduced spore germination, mycelial growth, and target gene expression. siRNA sequences were transfected into *P. fijiensis* mycelial fragments via the PEG‒LiCl method, which was then directly applied to ascospores. Furthermore, we evaluated the impact of siRNA transfection on fungal pathogenicity under controlled greenhouse conditions.

## Materials and methods

### Fungal material

Germination tests were conducted using *P. fijiensis* ascospores obtained from infected banana leaves collected at stages 5 and 6 of Black Sigatoka disease, according to the Fouré scale [34]. Ascospores were obtained via the discharge method described by Stover (1976), with slight modifications. Portions of banana leaves with symptoms of black sigatoka were cut into 2 × 2 cm squares and then attached to circular pieces of filter paper, with the top facing the paper. The pieces were wrapped in moist paper and incubated at 26 °C in plastic bags for 5 days in the dark. Next, the paper with the tissue was placed inside the lid of Petri dishes with the tissue facing the agar‒water plate (2%), but without making contact between them. Finally, the Petri dishes were incubated at 26 °C ± 1 for 60 min to allow the discharge of the ascospores. Ascospore discharge sites were located on Petri dishes via a light microscope and marked for further identification on the basis of their morphology and dimensions.

The evaluation of mycelial growth and gene expression levels via quantitative polymerase chain reaction (q-PCR) was carried out with *P. fijiensis* strain 080930 collected from Urabá-Antioquia, Colombia. This strain was previously isolated in Unidad de Biotecnología Vegetal de la Corporación para Investigaciones Biológicas (CIB), Medellín, Colombia, and characterized as having high aggressiveness and resistance to the fungicide propiconazole [35]. The infection assays were conducted using strain C139, which is sensitive to propiconazole. Before use, the strain was cultivated on potato dextrose agar (PDA, Becton Dickinson and Company, Le Pont de Claix, France) at 27 °C under red light for 15 days [36].

### Plant material and growth conditions

Banana plants of the cultivar Williams (triploid, AAA genome group) were obtained from the in vitro culture facilities of the Plant Biotechnology Unit of Universidad Católica de Oriente, Rionegro-Colombia. Three-month-old plants were kept under greenhouse conditions at 29 °C and a relative humidity above 95% with standard fertilization and irrigation practices and a 12 hr:12 hr light/dark photoperiod.

### siRNA design

*The PfFus3, Pf*AC and *PfCYP51* siRNA sequences were designed via the Rational siRNA design program [37] according to the protocol described by Birmingham, Anderson [38] and chemically synthesized via Bioneer Oligo Synthesis (Munpyeong-dong, Republic of Korea). The siRNAs homologous to the *Fus3* and *AC* genes were designed on the basis of the sequence of the corresponding *P. fijiensis*, *Fus3* and *AC* orthologs found in the *P. fijiensis* V. 2.0 genome database available at DOE Joint Genome Institute (JGI) (http://genome.jgi.doe.gov/Mycfi2/Mycfi2.home.html) gene models No. 51107 and 216002, respectively, with the sense strand being the 5' CGUCAACGCAGAUGGAUCA 3' and the antisense sequence being the 5' UGAUCCAUCUGCGUUGACG 3' for *Fus3* and the sense strand being 5' UGAAAGGCCUCUUGUUAUA 3' and the antisense sequence being the 5' UAUAACAAGAGGCCUUUCA 3' for the *AC* gene. The siRNA homologous to the *PfCYP51* gene was designed employing the sequence of the *PfCYP51*A gene published by [28] with accession number EF581093 (National Center for Biotechnology Information, NCBI), which has a sense sequence of 5' AGAUUAUGCUUACGUUCAA 3' and an antisense sequence of 5' UUGAACGUAAGCAUAAUCU 3'.

### Confirmation of siRNA cell entrance

To verify that the siRNA molecules entered the host cell, a fluorescein-labeled siRNA of 25 base pairs homologous to the *Fus3* gene was used with the sense sequence: 5' GAAAUGAAAUUGUUGCGCUACUUCA 3’ and antisense sequence: 5' UGAAGUAGCGCAACAAUUUCAUUUC 3’, chemically synthesized by Bioneer Oligo Synthesis. The mycelial suspension was exposed to the labeled sequences via the modified PEG/LiCl method reported by Moazeni, Khoramizadeh [17]. The cellular uptake of the siRNA sequences was detected via fluorescence microscopy via a Nikon Eclipse 80 microscope (Nikon, Tokyo, Japan) with a 40x objective lens and appropriate ﬂuorescence ﬁlters. One hundred cells were counted, and the percentage of cells showing fluorescence was estimated and considered the percentage of cells with siRNA entrance. This experiment was repeated three times.

### Effect of siRNA on ascospore germ tube growth

The effects of siRNA sequences homologous to the *PfFus3* gene on the ascospore germ tube growth of *P. fijiensis* were tested via four treatments. The treatments consisted of siRNA sequences resuspended in diethyl pyrocarbonate (DEPC)-treated water, siRNA sequences mixed with an equal volume of Lipofectamine (Invitrogen Life Technologies, Carlsbad, California, United States), Lipofectamine® alone and DEPC-treated water as controls. To determine the most effective concentration, siRNA sequence molecules homologous to *PfFus3* were tested at 50 nM, 75 nM, 100 nM and 150 nM. Twenty µl of siRNA solution was placed over ascospores discharged from fungicide-free infected leaves on Petri dishes containing agar-containing water (2%). The plates were then incubated for 48 hours, and photographs were taken to measure the length of the germ tube using ImageJ software [39]. The determination of the siRNA concentration that resulted in the best inhibition was repeated three times, with two replicates in each experiment.

The silencing of the *PfCYP51* gene was performed in the same way as that for the *PfFus3* assay but with 100 nM siRNA sequences. Ascospores were discharged over 2% agar-containing water supplemented with and without 0.5 mg/L propiconazole. This concentration was chosen on the basis of prior assays conducted in this study (S1 Fig in S1 File), where we determined that 0.5 mg/L propiconazole did not inhibit germ tube growth in the ascospores. In this way, we could ensure that the observed effects were due to the siRNA sequences and not fungicide action. The leaves used for the *PfCYP51* silencing experiments were collected from farms that were regularly sprayed with azole fungicides.

The siRNA sequences homologous to the *PfAC* gene were assayed without Lipofectamine. siRNAs at a concentration of 100 nM were dissolved in water treated with DEPC, and water treated with DEPC alone as a control was applied to the ascospores.

### Effect of siRNA on mycelial growth

A suspension of mycelial fragments of *P. fijiensis* strain 080930 grown on PDA was prepared via the use of a sterile scraping brush and distilled water. The suspension was filtered with sterile etamine cloth to obtain uniform mycelial fragments, and their concentration was adjusted to 1x10^6^ mycelial fragments/ml with sterile distilled water via a Neubauer chamber (1/10 mm deep, bright line-Boeco, Germany).

In a sterile 10 ml tube, 1 ml of this suspension was mixed with 2 ml of siRNAs at a final concentration of 100 nM. As a negative control, 1 ml of the suspension mixed with DEPC-treated water was used. The suspensions were incubated at 27 ^°^C for 24 h, after which 7 ml of sterile distilled water was added to each tube.

Additionally, the modified PEG transfection method previously reported by Moazeni, Khoramizadeh [17] with some modifications was used. Briefly, 1 ml of the mycelial suspension was centrifuged at 12000 rpm, resuspended in 500 µl of 100 mM LiCl, mixed gently, and centrifuged at 12000 rpm for 2 min. Afterwards, 240 µl of 50% PEG8000 and 36 µl of 1.0 M LiCl were added to the pellet. siRNA was then added to the suspension to achieve a final concentration of 100 nM. Finally, DEPC-treated water was added to a final volume of 360 µl. As a negative control, mycelial cells without siRNA were used. Each tube was vigorously vortexed for 5–10 s until the cell pellets were completely mixed. The cells were then subjected to heat shock in a water bath at 42 °C for 40 min, followed by centrifugation at 8,000 rpm for 30 s and resuspension in 500 µl of sterile distilled water.

Finally, the samples were inoculated into 96-well plates, each well containing 50 µl of treated mycelium plus 100 µl of Sabouraud broth (BBLTM Becton Dickinson, Sparks, MD, USA). The microplates were then incubated at 27 °C for 11 days and 14 days for direct transfection entry and modified-PEG/LiCl methods, respectively, and mycelial growth was measured by measuring the absorbance at 595 nm in a spectrophotometer (Bio-Rad model 550) [40]. For *PfCYP51* analysis, 50 µl of Sabouraud broth, 50 µl of siRNA-treated mycelium and 50 µl of propiconazole at 1.5 mg/L to a final concentration of 0.5 mg/L, which does not inhibit microbial growth (S2 Fig in S1 File), were added to each well of the microplate and compared to a control without propiconazole.

### Analysis of gene silencing via RT‒qPCR

Gene silencing in treated mycelia was confirmed by measuring gene expression via RT‒qPCR. For this purpose, 500 µl of a mycelial suspension treated as described above via the modified-PEG/LiCl transfection method was incubated in 1.5 ml tubes containing 1 ml of Sabouraud broth at 27 °C for 7 days before RNA isolation. Total RNA was purified via the commercial reagent Concert Plant RNA (Invitrogen, Carlsbad, California, United States). RNA was quantified via spectrophotometry (260/280 nm) via an ND-NanoDrop 2000 UV‒Vis Spectrophotometer (NanoDrop Technologies, USA) and visualized via 1% agarose gel electrophoresis under ultraviolet light via a Biometra BioDocAnalize transilluminator (Biometra, Germany). The gel was run at 100 V for 40 minutes using 0.5% TBE buffer and the loading buffer GelRed™ to determine quality and integrity. DNA contamination of the samples was removed via an RNase-free DNase I digestion kit (Fermentas, Waltham, United States). cDNA synthesis was performed via the Maxima First Strand cDNA Synthesis Kit for RT‒qPCR, following the manufacturer’s guidelines, with 100 ng of total RNA (Thermo Scientific MA, USA). RT‒qPCR was performed via the Maxima SYBR Green/ROX qPCR Master Mix Kit, following the manufacturer’s instructions, with specific primers (Table 1).

**Table 1:** Primers used for gene amplification in RT‒qPCR experiments. Primers used to amplify the *PfFus3*, *PfAC*, *PfCYP51*, and Actin genes for RT‒qPCR analysis in this study. The primers were designed to specifically target the corresponding genes and were used to assess gene expression levels in mycelial samples.

Gene	Primer Name	Sequence
*PfFus3*	Fus3F	5´ATCCACCGAGTCATCCGCACT 3´
	Fus3R	5´TCGGTGGAGGACATTGGCGGA 3´
*PfAC*	QACF	5´TATGCAGAATGGCCCGCTACCGCCT 3´
	QACR	5´TGGCTGAGGTGCAGGTGTTCGTGGT 3´
*PFCYP51*	CYP51F	5´CGCCAGTATTCGGCACAGATGTCG 3´
	CYP51R	5´TAACGTAGGACTGGAGGGCGGA 3´
Actin	ActinF	5´TTACGAGGTTTCGCTCTCC 3´
	ActinR	5´GATGTC GCGGACAATTTCAC3´

To determine primer amplification efficiency, a standard curve was generated using a four-point 1:10 serial dilution series, starting from 200 ng of genomic DNA. Amplification efficiencies for each primer pair were calculated from the slope of the corresponding standard curve. Relative gene expression levels were then quantified using the Pfaffl method [41], which incorporates individual primer efficiencies to improve the accuracy of the quantification compared to the conventional ΔΔCt method [42].

### Effect of siRNA transfection on pathogenicity

Banana plants (Musa spp.) cv. Williams (triploid, AAA genome group) were used to evaluate the effect of siRNA transfection on the pathogenicity of *Pseudocercospora fijiensis*. The plants were cultivated under greenhouse conditions at 28 ± 1 °C, with a relative humidity above 95% and a 12-hour light/dark photoperiod. The first three leaves of each plant were inoculated with mycelial fragments of P. fijiensis transfected with siRNA at a final concentration of 100 nM via the modified PEG/LiCl transfection method described above. The control plants were inoculated with mycelial fragments treated with 2% gelatin without siRNA transfection.

Infection levels were assessed by capturing images of infected leaves and analyzing them via ImageJ software [39]. The percentage of infection was calculated for each leaf. All the treatments, including the controls, followed a randomized complete block design on greenhouse benches. The experiment was conducted in triplicate.

### Statistical analysis

In the first assay with ascospores, data on germ tube length obtained from three *PfFus3* siRNAs (50 nM, 100 nM and 150 nM) were analyzed via analysis of variance (ANOVA). The Shapiro‒Wilk test was performed to verify normality, and the Levene test was used for homogeneity of variances; data that met these criteria were log-transformed. Data from the 75 nM group did not satisfy the assumption of variance homogeneity; thus, the data were analyzed via the Kruskal‒Wallis test.

Each repetition of *PfFus3* and *PfCYP51* gene silencing on ascospores at a 100 nM siRNA concentration was averaged and analyzed via a block design and an ANOVA test to determine differences between treatments. Shapiro‒Wilk tests were performed for normality, the Levene test was used for homogeneity of variance, and comparisons of means were performed via the Tukey test (P > 0.05).

Significant differences between treatments in the silencing of ascospores with siRNAs homologous to the *AC* gene and mycelia silencing were identified via Student’s t test, with tests of normality and homogeneity of variances performed as described above. Data that did not meet the assumptions were analyzed via the Wilcoxon signed-rank test. The RT-qPCR data, which were calculated via relative quantification, were analyzed via Student’s t test.

Repeated measures analysis via mixed models was performed for each infection lesion experiment. A model was fitted for each of the suggested covariances on the basis of the Bayesian information criterion (BIC). The autoregressive AR 1 model was chosen, and the corresponding ANOVA was evaluated. All analyses were performed via the computational package R [43].

Summary statistics and p-values for all tests, are provided in S1–S10 Tables in S2 File.

## Results

### Confirmation of siRNA entry into cells

**Fig 1:**
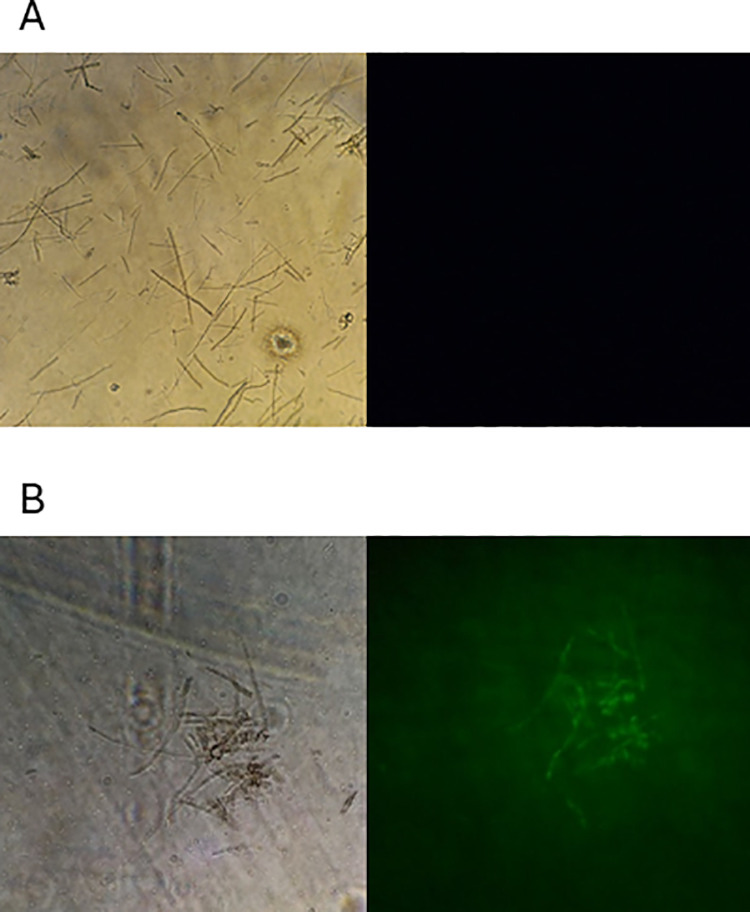
Fluorescence microscopy images of the cells treated with labeled *PfFus3* siRNAs. (A) Mycelium not exposed to siRNA but treated via the modified-PEG/LiCl method. (B) Mycelia exposed to siRNA via the modified-PEG/LiCl method under bright-field illumination (left) and mycelia exposed to siRNA via the modified-PEG/LiCl method under fluorescence (right).

Fluorescence microscopy was used to confirm the entrance of the siRNA molecules into the cells. Fluorescence was observed only in mycelial fragments exposed to labeled siRNA molecules. No fluorescence was observed for the nonlabeled controls. Approximately 62 ± 4.4% of mycelial fragments exposed to the labeled siRNA molecules via the modified PEG/LiCl method presented fluorescence (Fig 1).

### Effect of siRNA on ascospore germ tube growth

**Fig 2:**
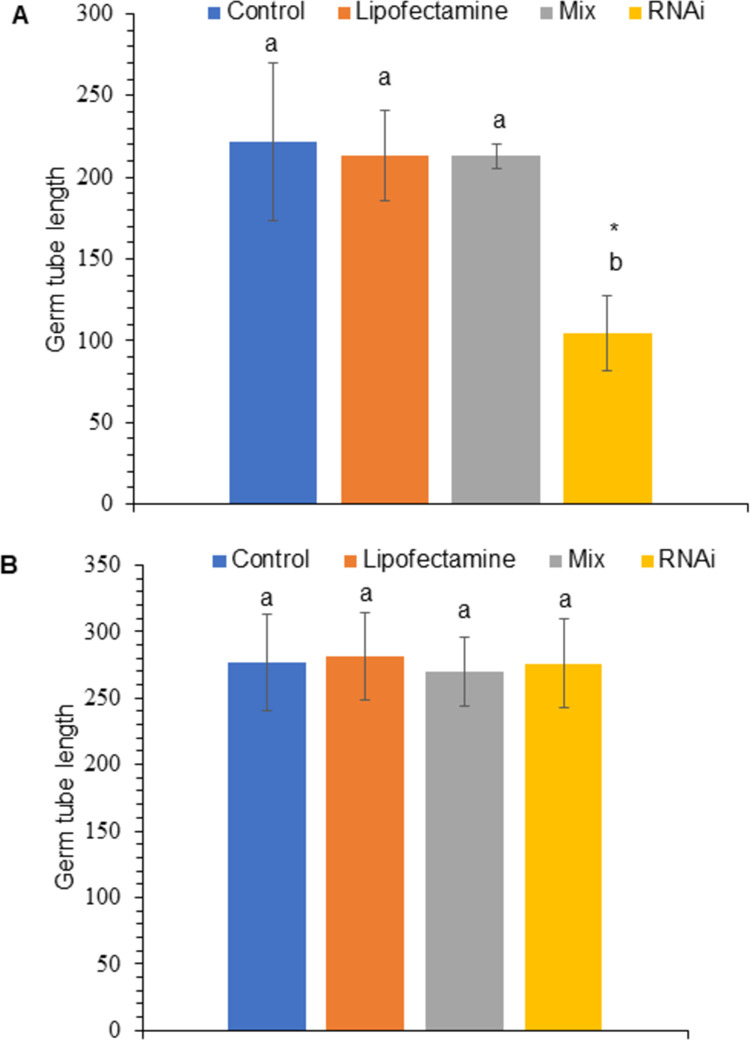
Germ tube length (μm) of ascospores treated with siRNAs homologous to *PfCYP51.* (A) Ascospores discharged on petri dishes containing water or agar supplemented with propiconazole at 0.5 mg/L. (B) Ascospores discharged on petri dishes containing water or agar without propiconazole. Treatments included siRNA sequences (RNAi), lipofectamine + siRNA (Mix), DEPC-treated water (control), and Lipofectamine at 100 nM of siRNA targeting the *PfCYP51* gene. At least 40 ascospores were measured per treatment, with 3 replications over time. The vertical bar indicates the standard deviation from the mean of 3 replicates. Different letters indicate significant differences between the values according to the Tukey test (* p < 0.05); identical letters indicate the absence of significance.

*PfCYP51* siRNA molecules at a concentration of 100 nM caused a 52.83% decrease in the germ tube length of ascospores discharged on agar‒water (2%) supplemented with propiconazole compared with that of control ascospores (p = 0.0241211; S1 Table in S2 File) (Fig 2A). Moreover, no significant differences in germ tube length were detected between ascospores discharged on agar water (2%) without propiconazole and the control (Fig 2B; S2 Table in S2 File).

**Fig 3:**
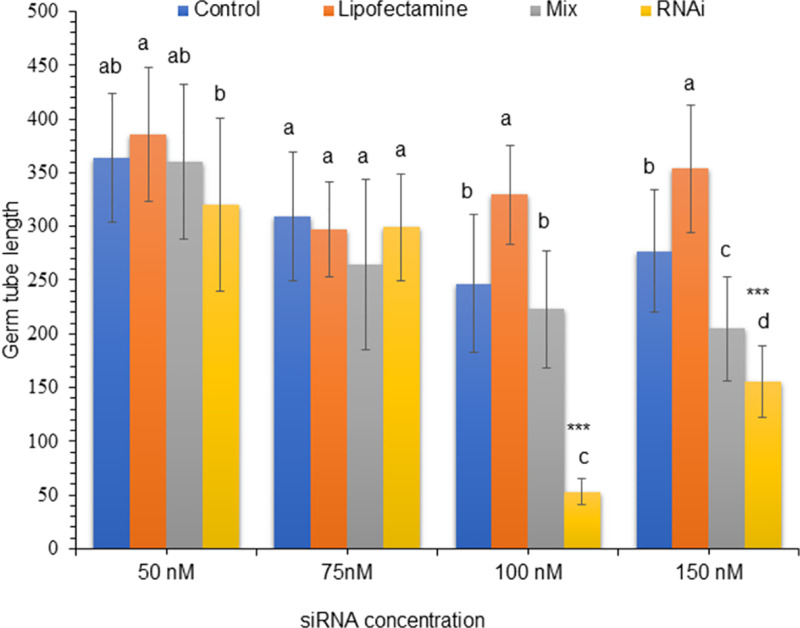
Germ tube length (μm) of ascospores treated with siRNAs homologous to *PfFus3.* The figure shows the germ tube length in μm of ascospores treated with siRNA sequences (RNAi), lipofectamine + siRNA sequences (Mix), DEPC-treated water (control), and Lipofectamine at concentrations of 50 nM, 75 nM, 100 nM, and 150 nM of siRNA targeting the *PfFus3* gene, which were measured after 48 hours. A total of 30 ascospores were measured for each treatment. The vertical bar indicates the standard deviation. Different letters indicate significant differences between the values according to the Tukey test (***p < 0.001); identical letters indicate the absence of significance.

**Fig 4:**
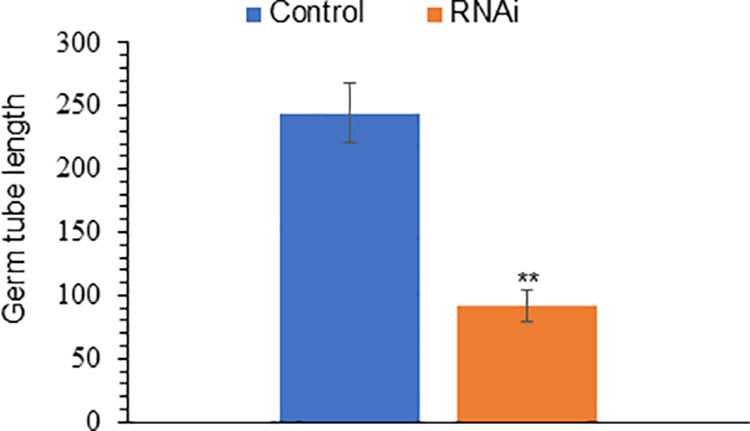
Germ tube length of ascospores treated with siRNAs homologous to *PfAC.* The figure shows the germ tube length of ascospores treated with siRNA homologous to the PfAC gene (RNAi) compared with those treated with DEPC-treated water (control). At least 40 ascospores were measured per treatment, with 3 replications throughout time. The vertical bar indicates the standard deviation from the mean of 3 replicates. **, Significantly different from the control according to the t test (p < 0.01).

The *PfFus3* siRNA sequence inhibited the germ tube length of *P. flijiensis* ascospores by 78.4% at 100 nM (p = 2e^-16^; S5 Table in S2 File) and 43.89% at 150 nM (p = 2e^-16^; S6 Table in S2 File) (Fig 3). When ascospores were treated with 150 nM but mixed with Lipofectamine®, there was only a slight decrease (26.1%) in spore germination (p < 0.0000011). Compared with the control ascospores, the germ tube growth of the ascospores treated with only Lipofectamine® did not significantly differ. No significant differences in germ tube length were observed when the ascospores were treated with 50 or 75 nM *PfFus3* siRNA (Fig 3; S3–S4 Tables in S2 File). In a similar manner to that of *PfFus3,* compared with the control, the siRNA homologous to the *PfAC* gene at a concentration of 100 nM inhibited the germ tube length of treated ascospores by 62.4% (p =0.002152; S7 Table in S2 File) (Fig 4).

### Effect of siRNA on the growth of mycelia

Mycelial growth after 14 days did not significantly differ from that of the untreated controls when the plants were directly exposed to the siRNA sequences. However, when mycelia were exposed to the siRNA sequences via the modified-PEG/LiCl method, differences between mycelia treated with siRNA sequences and untreated controls were observed for the *PfFus3*, *PfAC* and *PfCYP51* treatments. For the *PfCYP51* gene treatments, there were significant differences in the growth of mycelia with propiconazole compared with the nontreated controls, but no differences were found without the fungicide (Table 2; S8 Table in S2 File).

**Table 2:** The absorbance at 595 nm corresponds to mycelial growth via the modified PEG/LiCl transfection method. The values represent the average of forty absorbances measured per treatment for the PfAC, PfFus3 and PfCYP51 assays. Statistical analysis was performed with Student’s t test or the Wilcoxon test.

Gen	Replica	Absorbance to 595 nm		P- value
		Control n = 40	RNAi n = 40	
*PfAC*	R1	0.79 ± 0.36	0.56 ± 0.1	<0.001^b^
	R2	0.64 ± 0.098	0.45 ± 0.067	<0.001^a^
	R3	0.99 ± 0.417	0.67 ± 0.048	<0.001^b^
*PfFus3*	R1	0.93 ± 0.47	0.62 ± 0.22	<0.001^b^
	R2	0.96 ± 0.22	0.91 ± 0.27	<0.05^b^
	R3	0.96 ± 0.22	0.85 ± 0.15	<0.05^b^
*PfCYP51* with propiconazole to 0.5 mg/L	R1	0.13 ± 0.036	0.10 ± 0.005	<0.001^b^
	R2	0.25 ± 0.072	0.16 ± 0.045	<0.001^a^
	R3	0.23 ± 0.124	0.10 ± 0.013	<0.001^b^
*PfCYP51* without propiconazole	R1	0.40 ± 0.096	0.38 ± 0.13	0.0859^b^
	R2	0.61 ± 0.149	0.29 ± 0.062	<0.001^a^
	R3	0.61 ± 0.178	0.55 ± 0.197	0.1116^b^

^a^t test

^b^Wilcoxon signed rank test

### Analysis of gene silencing by RT‒qPCR

**Fig 5:**
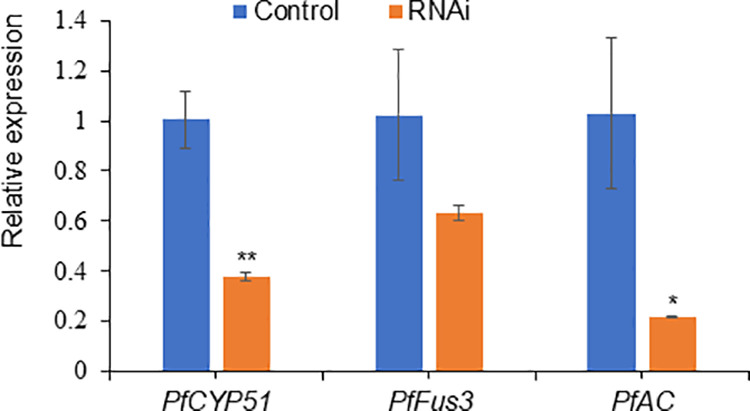
Relative gene expression levels in mycelia after siRNA treatment. The figure presents the relative gene expression levels in mycelia 7 days after exposure to the *PfCYP51*, *PfAC*, and *PfFus3* siRNAs. The values of the siRNA-treated mycelia were compared with those of the untreated control mycelia via Student’s t test; **, significantly different from the control (p < 0.01), *, significantly different from the control (p < 0.05).

RT‒qPCR was performed to measure the mRNA levels of the *PfAC*, *PfCYP51* and *PfFus3* genes at 7 days after the application of 100 nM siRNA molecules compared with those in untreated controls (Fig 5). The results revealed a decrease in the mRNA levels of the 3 analyzed genes in mycelia exposed to siRNA homologous sequences compared with their respective controls. There was a greater decrease in the mRNA levels of the *PfCYP51* and *PfAC* genes than in the *PfFus3* gene (Fig 5). Compared with those of the control (100%), the relative expression levels of the genes *PfCYP5*1, *PfAC*, and *PfFus3* were 0.38 (38%), 0.22 (22%), and 0.64 (64%), respectively.

### Effect of siRNA transfection on in vivo assays

**Fig 6:**
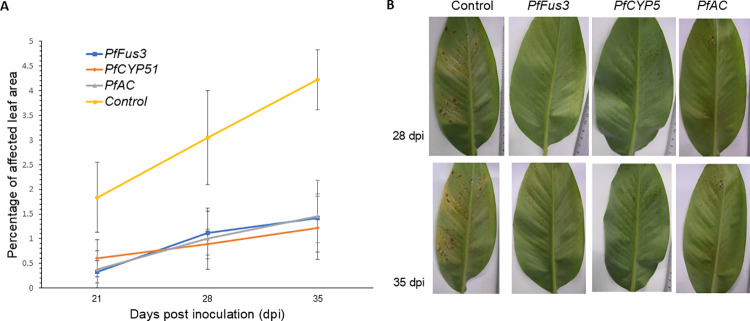
Percentage of affected leaf area in banana seedlings infected with mycelia transfected with siRNAs. (A) The figure shows the percentage of affected leaf area in banana seedlings (cv. Williams) infected with mycelia transfected with siRNAs targeting *PfFus3*, *PfCYP51*, and *PfAC*, compared with the control without transfection, at different days post inoculation. The bars represent the standard deviation. (B) Representative images of the abaxial side of plant leaves showing disease symptom progression at 28 and 35 days post-inoculation (dpi).

The effectiveness of treating *P. fijiensis* with siRNA sequences to reduce infection symptoms was evaluated. *P. fijiensis* mycelia transfected with siRNA sequences at a concentration of 100 nM via the PEG/LiCl method were inoculated onto the surface of banana leaves, and disease progression was assessed at 21, 28, and 35 days postinoculation. There was a significant reduction in the formation of local necrotic lesions in plants sprayed with mycelia transfected with siRNA *PfFus3*, *PfCYP51*, and *PfAC* sequences during the same time periods (Fig 6). These differences were significant between the treatments (p < 0.0001; S10 Table in S2 File). For the siRNAs *PfFus3* and *PfCYP51*, infected leaves presented percentages of affected leaf area 3 and 3.5 times lower than those of the control, respectively. Similarly, concerning the adenylate cyclase gene, the infection percentage was 2.9 times lower than that of the control (p < 0.0001; S10 Table in S2 File).

## Discussion

The molecular tools available to study gene function in fungi are limited, particularly because homology-dependent recombination for gene knockout studies is technically difﬁcult to achieve for some species, such as *P. fijiensis*. Thus, RNA silencing has become an important alternative, especially in cases where a complete gene knockout causes a lethal phenotype [44]. However, it is necessary to develop adequate methods for siRNA gene silencing in *P. fijiensis* as few studies have been performed on this important pathogen. In the present study, we demonstrated that siRNA molecules directly applied to *P. fijiensis* enter fungal cells and induce gene silencing. With some improvements, this methodology could be used as a tool for gene function studies that ultimately will allow for screening new control targets and possibly as a direct treatment for Black Sigatoka disease, as has been shown in other plant pathosystems [14].

Posttranscriptional gene silencing in *P. fijiensis* has been previously reported [24]. In that study, the in vitro antifungal activities of a set of synthetic dsRNA molecules on the germination of *P. ﬁjiensis* spores were evaluated by determining the number of colony-forming units (CFUs). Compared with the control, direct application of dsRNA sequences inhibited colony growth in spores. Although the results of that study were favorable, there was no direct evidence of mRNA expression levels of the gene target or that the dsRNA molecules entered the fungal cells. In the present study, the entry of siRNAs into *P. fijiensis* mycelial cells was confirmed via the use of labeled siRNAs and fluorescence microscopy. However, fluorescence was not observed in any of the mycelia, suggesting limited efficiency of the process. This phenomenon has also been observed in other fungi. For example, in *Candida albicans*, the efficiency of siRNA entry into yeast cells has been reported to be low because of the direct delivery of siRNA sequences from the medium [17].

Differences in the entry of siRNAs into cells may be due to the characteristics of each organism, which could favor the entrance of molecules, such as charge, permeability, and/or rupture of the cell wall and membrane. In filamentous fungi, the cell wall has been reported as the main obstacle to successful gene transformation. Protoplasts obtained from young hyphae have been shown to be suitable for transformation. However, obtaining protoplasts is a cumbersome and expensive procedure, and their low rate of regeneration limits their application [26,45]. On the other hand, at certain Li+ concentrations, membrane permeability increases, allowing exogenous DNA to pass through the cell wall and thereby improving transformation efficiency [26]. Our study demonstrated that lithium treatment facilitates the entry of siRNA sequences into fungal cells. After establishing the siRNA entry method, we assessed the potential effects of the siRNA sequences on growth and pathogenicity related to the silencing of the *PfCYP51*, *PfFus3,* and *PfAC* genes.

The silencing of the *PfCYP51* gene effectively reduced resistance to the DMI fungicide propiconazole, supporting the hypothesis that resistance to DMIs is at least partially driven by the overexpression of *PfCYP51*. Previous studies in *P. fijiensis* have shown that resistance to DMIs involves both the presence of repetitive elements in the promoter region of *PfCYP51*, which leads to overexpression of the gene, and mutations in the coding region of *PfCYP51* [28,29,46]. These mutations result in reduced sensitivity to DMIs and cause nonsynonymous amino acid substitutions at substrate recognition sites (SRSs) in the enzyme. Therefore, while the overexpression of *PfCYP51* contributes to resistance, it is not the only mechanism involved. Further research is needed to evaluate whether RNAi-mediated silencing of *PfCYP51* can effectively overcome azole resistance caused by both overexpression and mutation of the coding region.

RNAi technology may constitute a way to combat fungicide resistance to DMIs in *P. fijiensis* as well as to directly combat the disease. This could be achieved by directly applying siRNA sequences incorporated into fungicides or by using host-induced gene silencing via transgenic plants that express siRNA sequences homologous to the *PfCYP51* gene. It has been reported that spraying wheat plants with dsRNA targeting the Myosin 5 gene of *F. asiaticum* via RNAi confers high levels of resistance against this fungus, with a significant reduction in resistance to phenamacril, a fungicide that inhibits myosin [47]. Additionally, an effective reduction in symptoms has been reported in Arabidopsis and barley expressing dsRNA sequences directed against *CYP51* from *F. graminearum* [14,48].

siRNA molecules homologous to *PfFus3* reduced the average germ tube length, suggesting that this gene is important for the growth and development of *P. fijiensis.* This finding is consistent with the role reported for this family of serine protein kinases [31,49,50]. Additionally, our results align with a study in which RNAi-mediated gene silencing of *PfFus3* in *P. fijiensis* transformants resulted in significantly lower gene expression, reduced virulence, and decreased invasive growth in infected banana leaf tissues [21].

Similar to the sequences of the siRNAs *PfCYP51* and *PfFus3*, the siRNA sequences homologous to the *PfAC* gene were able to decrease mycelial growth and the length of the germ tube from the ascospores of *P*. *fijiensis*. *AC* is an enzyme that catalyzes the formation of cyclic AMP from ATP. This is highly important given that cAMP acts as a second messenger in the signal transduction of numerous biological processes [33]. This enzyme has been investigated in several funga1 species. In *Magnaporthe grisea,* the deletion of this gene resulted in a reduction in vegetative growth, conidiation and conidial germination. *M. grisea* transformants are unable to form appressoria on an inductive surface, are not able to penetrate susceptible rice leaves, are sterile and do not produce perithecia [51]. Similarly, the suppression of cAMP levels in *Sclerotinia sclerotiorum* resulted in decreased growth rates and attenuated virulence [52]. In the phytopathogen *Ustilago maydis*, mutants of the *AC* gene are not pathogenic and exhibit constitutive filamentous growth due to an inability to regulate the dimorphic growth cycle [53]. In *Botrytis cinerea*, disruption of the *AC* gene resulted in reduced vegetative growth, low intracellular cAMP levels, slow lesion development, and a lack of *in planta* sporulation [54].

As described in previous studies, *Fus3, CYP51*, and *AC* have been identified as important genes involved in regulating pathogenicity in other fungal pathogens. In this study, we investigated the role of *Fus3, CYP51*, and *AC* in the pathogenicity of *P. fijiensis* in banana plants. Compared with plants inoculated with mycelia without siRNA, those with silenced *PfFus3*, *PfCYP51*, and *PfAC* presented reduced virulence, characterized by a lower efficiency of plant infection and fewer necrotic symptoms, in the susceptible cultivar ‘Williams’.

Lipofectamine® has been successfully used to deliver synthetic siRNA into fungal protoplasts in a short number of studies [18,55]. However, in our study, the growth inhibition observed for both PfCYP51 and PfFus3 siRNAs was greater when applied alone than when mixed with Lipofectamine®. Although this pattern could suggest more effective gene silencing without the reagent, we did not directly measure gene expression in these specific conditions. One partial explanation for this observation is that Lipofectamine® may require the absence of the fungal cell wall (i.e., the use of protoplasts) to effectively mediate siRNA uptake, whereas our experiments were conducted with intact cells.

RT‒qPCR measurements revealed that the mRNA levels of the *PfAC*, *PfCYP51* and *PfFus3* genes decreased after 7 days of treatment with siRNA molecules homologous to the corresponding *PfAC*, *PfCYP51* and *PfFus3* genes. In several fungi, RNAi silencing is activated within a few hours of siRNA sequence entry and persists for a few days. However, the time of induction of silencing and the duration of its effect vary among the different fungal species. For example, in *Aspergillus fumigatus,* the use of siRNA molecules against the *odcA* and *pyrG* genes resulted in a decrease in mRNA levels from 30–60% after 16 hours of exposure to the treatment compared with the corresponding controls [19]. In the oomycete filamentous pathogen *Phytophthora infestans*, RNAi silencing via dsRNA sequences specific to the *gfp*, *inf1*, and *cdc* 14 genes resulted in a decrease in mRNA levels up to 12 and 15 days after exposure to dsRNA (17 days for *gfp*) [55]. These studies show that the duration of active silencing is different in each organism or in the expression levels of each target gene.

In conclusion, in the present study, the direct application of siRNA sequences induced the silencing of genes associated with growth, pathogenicity and fungicide resistance in *P. fijiensis*. Additionally, we adapted an efficient method for efficient silencing of mycelia on the basis of the use of LiCl and PEG. This technique has potential for Black Sigatoka disease control; however, further research is needed to select and optimize specific conditions for each target gene. In addition, to the best of our knowledge, the results obtained in the present research show for the first time that the use of *PfCYP51* siRNA reduces the level of resistance to propiconazole under in vitro conditions.

## Supporting information

S1 File S1–S2 Figs, which present the results of the analysis of germ tube length and mycelial growth of *Pseudocercospora fijiensis* under fungicide selection pressure with different concentrations of propiconazole. (DOCX)

S2 File S1–S10 Tables, which present the results of statistical analyses performed to evaluate the effectiveness of gene silencing mediated by siRNA in *Pseudocercospora fijiensis.* (DOCX)
