# Supplementary material for: RNA-mediated gene silencing can reduce azole resistance, growth and pathogenicity in Pseudocercospora fijiensis
Source: PLoS One. 2025 Jun 5;20(6):e0325057. doi: 10.1371/journal.pone.0325057 (PMC12140194; doi:10.1371/journal.pone.0325057)
Supplement: S1 File — (DOCX) [file pone.0325057.s001.docx]

**
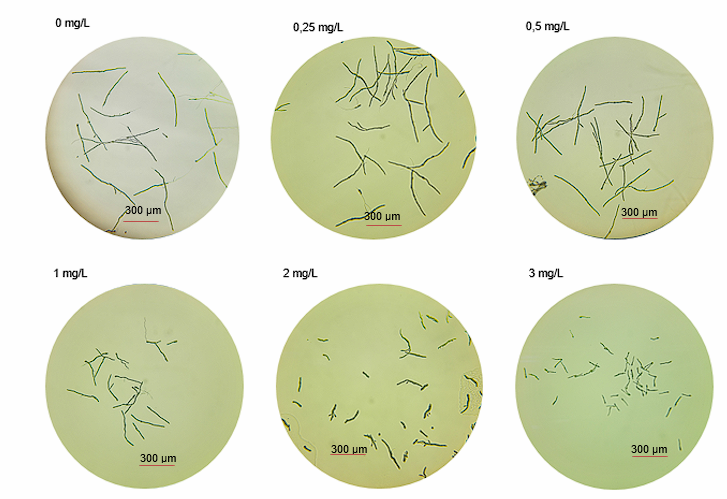
**

**Figure S1:** Germ tube length of *Pseudocercospora fijiensis* ascospores after 48 hours of incubation under fungicide selection pressure on PDA medium supplemented with different concentrations of propiconazole.


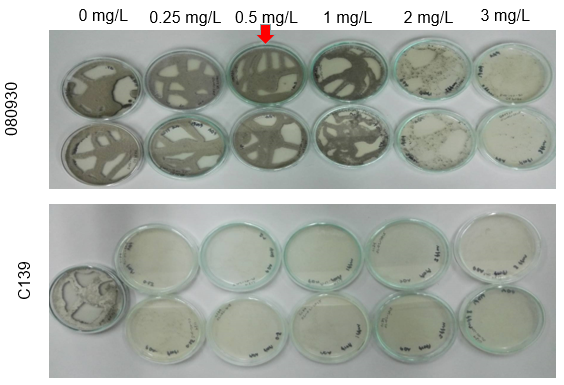


**Figure S2:** Mycelial growth of Pseudocercospora fijiensis strains C139 (sensitive) and C86 (resistant) after incubation on PDA medium supplemented with different concentrations of propiconazole.
