# Supplementary material for: RNA-mediated gene silencing can reduce azole resistance, growth and pathogenicity in Pseudocercospora fijiensis
Source: PLoS One. 2025 Jun 5;20(6):e0325057. doi: 10.1371/journal.pone.0325057 (PMC12140194; doi:10.1371/journal.pone.0325057)
Supplement: S2 File — (DOCX) [file pone.0325057.s002.docx]

**Supplementary material: Statistical analyses**

This document presents the statistical analyses performed to evaluate the effectiveness of gene silencing mediated by small interfering RNA (siRNA) in *Pseudocercospora fijiensis*.

**S1 Tables:** ANOVA results (A) and Tukey test results (B) for germ tube length (μm) of ascospores treated with 100 nM siRNA targeting the PfCYP51 gene, discharged on medium supplemented with 0.5 ppm propiconazole.

1. ANOVA results

|  | Df | Sum Sq | Mean Sq | F-value | p-value |
| --- | --- | --- | --- | --- | --- |
| Treatments | 3 | 28038 | 9346 | 7.694 | 0.0177 |
| Blocks | 2 | 30 | 15 | 0.013 | 0.9876 |
| Residuals | 6 | 7288 | 1215 |  |  |

1. Tukey's test result

|  | diff | lvr | upr | p adj |
| --- | --- | --- | --- | --- |
| Lipofectamine-control | -8.3500 | -106.86071 | 90.160710 | 0.9903188 |
| Mix-control | -8.9000 | -107.41071 | 89.610710 | 0.9883521 |
| RNAi-control | -117.0833 | -215.59404 | -18.572623 | 0.0241211 |
| Mix-Lipofectamine | -0.5500 | -99.06071 | 97.960710 | 0.9999971 |
| RNAi-lipofectamine | -108.7333 | -207.24404 | -10.222623 | 0.0332694 |
| RNAi-Mix | -108.1833 | -206.69404 | -9.672623 | 0.0339939 |

**S2 Table:** ANOVA results for germ tube length (μm) of ascospores treated with 100 nM siRNA targeting the PfCYP51 gene, discharged on medium without propiconazole.

|  | Df | Sum Sq | Mean Sq | F-value | p-value |
| --- | --- | --- | --- | --- | --- |
| Treatments | 3 | 201 | 67.1 | 0.186 | 0.9023 |
| Blocks | 2 | 6164 | 3082.2 | 8.536 | 0.0176 |
| Residuals | 6 | 2166 | 361.1 |  |  |

**S3 Tables:** ANOVA results (A) and Tukey test results (B) for germ tube length (μm) of ascospores treated with 50 nM siRNA targeting the PfFus3 gene.

1. ANOVA results

|  | Df | Sum Sq | Mean Sq | F-value | p-value |
| --- | --- | --- | --- | --- | --- |
| Treatments | 3 | 26.25 | 8.750 | 4.547 | 0.00478 |
| Residuals | 113 | 217.48 | 1.925 |  |  |

1. Tukey's test result

|  | diff | lvr | upr | p adj |
| --- | --- | --- | --- | --- |
| Lipofectamine-control | 0.42820197 | -0.5302705 | 1.38667442 | 0.6500838 |
| Mix-control | -0.07880952 | -1.0294024 | 0.87178339 | 0.9964117 |
| RNAi-control | -0.87547619 | -1.8260691 | 0.07511672 | 0.0825582 |
| Mix-Lipofectamine | -0.50701149 | -1.4490889 | 0.43506593 | 0.4998666 |
| RNAi-lipofectamine | -1.30367816 | -2.2457556 | -0.36160073 | 0.0025619 |
| RNAi-Mix | -0.79666667 | -1.7307263 | 0.13739294 | 0.1229647 |

**S4 Table:** Kruskal test results for germ tube length (μm) of ascospores treated with 75 nM siRNA targeting the PfFus3 gene.

| Kruskal-Wallis chi-squared | Df | P-Value |
| --- | --- | --- |
| 5.9792 | 3 | 0.1126 |

**S5 Tables:** ANOVA results (A) and Tukey test results (B) for germ tube length (μm) of ascospores treated with 100 nM siRNA targeting the PfFus3 gene.

1. ANOVA results

|  | Df | Sum Sq | Mean Sq | F-value | p-value |
| --- | --- | --- | --- | --- | --- |
| Treatments | 3 | 60.3 | 20.099 | 381.9 | 2e-16 |
| Residuals | 116 | 6.1 | 0.053 |  |  |

1. Tukey's test result

|  | diff | lvr | upr | p adj |
| --- | --- | --- | --- | --- |
| Lipofectamine-control | 0.31376403 | 0.1593717 | 0.46815637 | 0.0000034 |
| Mix-control | -0.09798281 | -0.2523752 | 0.05640953 | 0.3526682 |
| RNAi-control | -1.52698428 | -1.6813766 | -1.37259194 | 0.0000000 |
| Mix-Lipofectamine | -0.41174685 | -0.5661392 | -0.68635597 | 0.0000000 |
| RNAi-lipofectamine | -1.84074831 | -1.9951407 | -1.68635597 | 0.0000000 |
| RNAi-Mix | -1.42900147 | -1.5833938 | -1.27460913 | 0.0000000 |

**S6 Tables:** ANOVA results (A) and Tukey test results (B) for germ tube length (μm) of ascospores treated with 150 nM siRNA targeting the PfFus3 gene.

1. ANOVA results

|  | Df | Sum Sq | Mean Sq | F-value | p-value |
| --- | --- | --- | --- | --- | --- |
| Treatments | 3 | 269.2 | 89.74 | 88.17 | 2e-16 |
| Residuals | 116 | 118.1 | 1.02 |  |  |

1. Tukey's test result

|  | diff | lvr | upr | p adj |
| --- | --- | --- | --- | --- |
| Lipofectamine-control | 1.5300000 | 0.8509863 | 2.209014 | 0.0000002 |
| Mix-control | -1.4466667 | -2.1256804 | -0.767653 | 0.0000011 |
| RNAi-control | -2.4333333 | -3.1123470 | -1.754320 | 0.0000000 |
| Mix-Lipofectamine | -2.9766667 | -3.6556804 | -2.297653 | 0.0000000 |
| RNAi-lipofectamine | -3.9633333 | -4.6423470 | -3.284320 | 0.0000000 |
| RNAi-Mix | -0.9866667 | -1.6656804 | -0.307653 | 0.0013695 |

**S7 Table:** t-Test results for germ tube length (μm) of ascospores treated with 100 nM siRNA targeting the PfAC gene.

| t | df | p-value |
| --- | --- | --- |
| 10.013 | 2.9895 | 0.002152 |

**S8 Table:** Statistical analysis of mycelial growth using the modified-PEG/LiCl transfection method.

| Gen | Replica | t-Test | | | Wilcoxon Singned Ranks Test | |
| --- | --- | --- | --- | --- | --- | --- |
|  |  | t | df | p-value | w | p-value |
| *PfAC* | R1 |  |  |  | 1287 | 1.056e-06 |
|  | R2 | 10.302 | 68.896 | 1.372e-15 |  |  |
|  | R3 |  |  |  | 1375 | 3.539e-09 |
| *PfFus3* | R1 |  |  |  | 1164 | 0.0003644 |
|  | R2 |  |  |  | 1020 | 0.03467 |
|  | R3 |  |  |  | 1076 | 0.008025 |
| *PfCYP51* with propiconazole to 0.5 mg/L | R1 |  |  |  | 1209.5 | 8.289e-05 |
|  | R2 | 6.149 | 76.66 | 3.267e-08 |  |  |
|  | R3 |  |  |  | 1397 | 9.464e-09 |
| *PfCYP51* without propiconazole | R1 |  |  |  | 979 | 0.0859 |
|  | R2 | 14.906 | 77.989 | 2.2e-16 |  |  |
|  | R3 |  |  |  | 966 | 0.1116 |

**S9 Table:** t-Test results of relative quantification in RT-qPCR data.

| Gen | t-test | | |
| --- | --- | --- | --- |
|  | t | df | p-value |
| *PfCYP51* | 9.3876 | 2.1051 | 0.009443 |
| *PfFus3* | 2.5639 | 2.0529 | 0.1212 |
| *PfAC* | 4.6378 | 2.0003 | 0.04347 |

**S10 Tables:** Statistical analysis of infection lesion data. (A) Model selection. (B) ANOVA results. (C) Pairwise comparisons.

1. **Model selection**

| **Model** | **df** | **AIC** | **BIC** | **logLik** |
| --- | --- | --- | --- | --- |
| mod.SC | 15 | 118.6413 | 142.3941 | -44.32065 |
| mod.AR1 | 15 | 106.8342 | 130.5870 | -38.41711 |

*The AR(1) model was chosen due to a lower BIC value.*

1. **ANOVA results for the selected model (mod.AR1)**

| Effect | numDF | denDF | F-value | p-value |
| --- | --- | --- | --- | --- |
| Intercept | 1 | 33 | 103.19115 | <.0001 |
| Treatment | 3 | 33 | 12.09977 | <.0001 |
| Time | 2 | 33 | 30.87849 | <.0001 |
| Treatment:Time | 6 | 33 | 2.06969 | 0.0839 |

Significant interaction detected.

1. **Pairwise treatment comparisons**

| Contrast | Estimate | SE | df | t-ratio | p-value |
| --- | --- | --- | --- | --- | --- |
| *PfAC* - CT | -2.09033 | 0.305 | 33 | -6.857 | <.0001 |
| *PfAC* - *PfCYP51* | 0.03924 | 0.305 | 33 | 0.129 | 1.0000 |
| *PfAC* - *PfFus3* | -0.00533 | 0.358 | 33 | -0.015 | 1.0000 |
| CT - *PfCYP51* | 2.12957 | 0.358 | 33 | 5.942 | <.0001 |
| CT - *PfFus3* | 2.08501 | 0.366 | 33 | 5.698 | <.0001 |
| *PfCYP51* - *PfFus3* | -0.04456 | 0.305 | 33 | -0.146 | 1.0000 |
